# Juvenile nephropathy resembling human nephronophthisis–medullary cystic kidney disease in a 9‐month‐old domestic shorthaired cat

**DOI:** 10.1111/jsap.13863

**Published:** 2025-04-02

**Authors:** N. Goody, J. Poldy, A. Malbon, T. Morrison, I. Montanes‐Sancho, S. Dancer, D. Gunn Moore

**Affiliations:** ^1^ Hospital for Small Animals, Royal (Dick) School of Veterinary Studies The University of Edinburgh, Easter Bush Campus Midlothian UK; ^2^ Veterinary Pathology Unit, Royal (Dick) School of Veterinary Studies The University of Edinburgh, Easter Bush Campus Midlothian UK

A 9‐month‐old male neutered domestic shorthair cat presented with a 2‐month history of progressive lethargy, inappetence, and polydipsia. Weekly weight measurements of the patient and its littermate showed initial reduced weight gain, then weight loss (Fig 1A). The initial bloodwork revealed elevated creatinine (516 μmol/L; reference interval [RI] 22–162 μmol/L), urea (40.2 mmol/L; RI 2.8–9.8 mmol/L), potassium (5.6 mmol/L; RI 4–5 mmol/L), and phosphate (3.6 mmol/L; RI 1.4–2.5 mmol/L), with a haematocrit of 18% (RI 30%–45%). Urine specific gravity was 1.006 (RI > 1.035), without proteinuria, and sterile on culture. Ultrasonography revealed marked bilateral nephropathy (Fig 1B), seen as mild renomegaly (left length 4.6 cm, right 4.8 cm; RI < 4.5 cm), mild pyelectasia (5 mm bilaterally; RI < 3.5 mm), marked diffuse cortical and medullary hyperechogenicity, and numerous small, rounded anechoic cysts along the corticomedullary junction (CMJ). Each renal resistive index was within the normal range (right 0.58, left 0.62. Imaging differentials prioritized renal maldevelopment, acute kidney injury, or degeneration following nephrotoxicosis. Failure to respond to supportive therapy and acute deterioration warranted euthanasia. Necropsy confirmed numerous dilated cysts (up to 1 mm), bilaterally along the CMJ of the kidneys (Fig 1C). Histologically, these were markedly dilated corticomedullary tubules. Numerous oxalate crystals, chronic infarcts, and occasional atrophic and cystic glomeruli with pericapsular fibrosis were also present (Fig 1D). Although feline polycystic kidney disease (PKD) cysts are described at the CMJ, the clinical course and histological appearance were atypical of PKD. Juvenile onset, corticomedullary localization of the tubular cysts, and relatively limited glomerular lesions are features of human nephronophthisis‐medullary cystic kidney disease. These features have been rarely reported  in veterinary patients.

**FIG 1:**
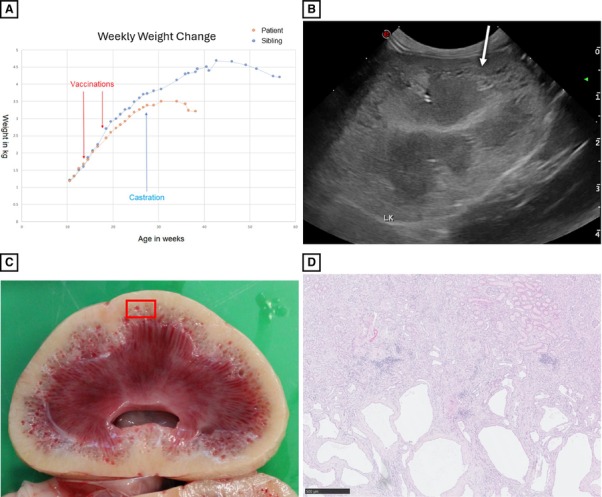
(A) Temporal changes in the patient's weekly body weight, compared to his sibling, demonstrating chronic reduction in weight gain progressing to weight loss. Both kittens were given their vaccines and were castrated at the same time as each other. The sibling developed the same signs at 12 months old, deteriorated, and was euthanised; renal histopathology was the same. (B) Sagittal ultrasonographic image of the left kidney depicting diffuse renal hyperechogenicity and numerous small anechoic cysts along the corticomedullary margin (white arrow). (C) Macroscopic examination of a sagittally sectioned unfixed kidney shows innumerable cystic cavities, up to 1 mm in diameter, along the corticomedullary interface (white arrow). (D) Markedly dilated tubules, lined by attenuated epithelium, are present at the corticomedullary junction. There is variable interstitial fibrosis and lymphocytic infiltration, as well as occasional cystic glomerular atrophy and scattered oxalate crystals (not shown). Haematoxylin and eosin stain. Scale bar = 500 μm.

## Author contributions

**N. Goody:** Data curation (equal). **J. Poldy:** Data curation (equal). **A. Malbon:** Data curation (equal). **T. Morrison:** Data curation (equal). **I. Montanes‐Sancho:** Data curation (equal). **S. Dancer:** Data curation (equal). **D. Gunn Moore:** Data curation (equal).

